# Stimulated by retinoic acid gene 8 (STRA8) interacts with the germ cell specific bHLH factor SOHLH1 and represses c‐KIT expression in vitro

**DOI:** 10.1111/jcmm.16087

**Published:** 2020-11-25

**Authors:** Maria Giovanna Desimio, Eleonora Cesari, Maria Sorrenti, Massimo De Felici, Donatella Farini

**Affiliations:** ^1^ Department of Biomedicine and Prevention Section of Histology and Embryology University Tor Vergata Rome Italy; ^2^ Department of Neuroscience Section of Human Anatomy Catholic University of the Sacred Heart Rome Italy; ^3^ Present address: Unit of Congenital and Perinatal Infection Academic Department of Pediatrics Bambino Gesù Childrens’ Hospital‐Scientific Institute for Research and Healthcare (IRCCS) Rome Italy

**Keywords:** c‐KIT, EBox‐mediated transcriptional regulation, meiosis, SOHLH1, STRA8

## Abstract

STRA8 (Stimulated by Retinoic Acid Gene 8) controls the crucial decision of germ cells to engage meiotic division up and down‐regulating genes involved in the meiotic programme. It has been proven as an amplifier of genes involved in cell cycle control and chromosome events, however, how STRA8 functions as negative regulator are not well understood. In this study, we demonstrate that STRA8 can interact with itself and with other basic Helix‐Loop‐Helix (bHLH) transcription factors through its HLH domain and that this domain is important for its ability to negatively interfere with the Ebox‐mediated transcriptional activity of bHLH transcription factors. Significantly, we show that STRA8 interacts with TCF3/E47, a class I bHLH transcription factors, and with SOHLH1, a gonadal‐specific bHLH, in male germ cells obtained from prepuberal mouse testis. We demonstrated that STRA8, indirectly, is able to exert a negative control on the SOHLH1‐dependent stimulation of c‐KIT expression in late differentiating spermatogonia and preleptotene spermatocytes. Although part of this results were obtained only ‘in vitro’, they support the notion that STRA8 interacting with different transcription factors, besides its established role as ‘amplifier’ of meiotic programme, is able to finely modulate the balance between spermatogonia proliferation, differentiation and acquisition of meiotic competence.

## 1 INTRODUCTION

Retinoic Acid (ATRA) has been shown to regulate several biological processes, in particular during embryonic development in mammals.^1^ Concerning reproduction, ATRA was shown to be crucial for the differentiation of gonocytes and spermatogonia^2^ and to induce the onset of meiosis in female primordial germ cell (PGCs)/oogonia and in spermatogonia from puberty onward.^3^ STIMULATED BY RETINOIC ACID GENE 8 (STRA8) protein has been proposed as the molecular effector of such promoting ATRA action. In line with this concept, mouse *Stra8* gene, originally identified in Embryonal Stem (ES) and Embryonal Carcinoma (EC) cells after ATRA treatment^4^, ^5^ is expressed at relatively high levels in male and female pre‐meiotic germ cells.^6^, ^7^, ^8^ Moreover, *Stra8* expression appears indispensable for the mitotic/meiotic switch in female PGCs and in male germ cells.^6^, ^9^, ^10^, ^11^ Besides, STRA8 promotes spermatogonial differentiation.^12^ Despite the well‐demonstrated importance of STRA8 in both sexes, its molecular function/s. has only recently been highlighted.^13^, ^14^ First described as a cytoplasmic protein,^5^ it has been successively demonstrated that it actually shuttles between the nucleus and cytoplasm.^15^ It has also been shown that STRA8 can bind DNA and possess a transcriptional activation domain in the C‐terminal region of its molecule.^13^, ^14^, ^15^, ^16^ Very recently, it has been demonstrated that STRA8 directly up‐regulates a large set of genes by binding to their promoter in the male germ cells at the preleptotene stage.^12^ Ishiguro et al, (2020)^14^ identified a STRA8‐interacting protein, MEIOSIN that is also required for the mitosis‐meiosis switching. Both MEIOSIN and STRA8 possesses a conserved region of the protein containing a Helix‐Loop‐Helix (HLH) domain. This is a homo‐ or hetero‐dimerization domain that characterizes the large family of HLH transcription factors and consists of highly conserved amphipathic helices separated by a loop of variable length and sequence.^17^ HLH proteins, through the regulation of gene expression, orchestrate cell cycle, cell lineage commitment and cell differentiation.^18^ Different groups of HLH proteins can be distinguished based on the presence or absence of additional functional domains.^17^, ^18^ Almost all HLH proteins possess a region of basic residues adjacent to the HLH domain that facilitates binding to DNA at a specific sequence motif known as EBox (CANNTG) or at the related NBox (CACNAG).^17^ X‐ray crystallographic analyses of bHLH proteins have defined the invariant basic sequence ER*X*R as the determinant for EBox recognition.^19^ Class I bHLH factors, also known as E proteins, are encoded by *Tcf3*/*Tcfe2a* (*E12, E47*), *Tcf4* and *Tcf12 (HEB)* genes. These proteins are widely expressed whereas class II bHLH proteins, which include members such as MYOD, MYOGENIN, NEUROD/BETA2, MASH and HAND, show a tissue‐restricted or lineage‐specific pattern of expression. Dimerization is essential for the DNA‐binding and transcriptional activity of these factors. In general, class II bHLH proteins form heterodimers with class I bHLH proteins, although they can also operate as homodimers. Most HLH proteins are transcriptional activators and contain distinct activation domains that can be physically separated from their DNA‐binding domains. Beside, other HLH proteins function as transcriptional repressors, for example, HAIRY, HES and STRA13/DEC2 and have an unusual DNA‐binding domain in which a proline is present in the basic region which gives specificity for NBox binding.^20^

A distinct subfamily of HLH proteins, the ID proteins (Inhibitor of DNA binding), lacks the basic region adjacent to the HLH domain which is essential for DNA binding. In mammalian cells, the ID family contains four proteins (ID1‐4)^21^ that affect the balance between cell growth and differentiation by negatively regulating the function of bHLH transcription factors.^22^ ID proteins bind to both class I and class II bHLH proteins and inhibit their ability to bind DNA through the formation of inactive heterodimers.^22^ Consequently, the expression of genes that possess the EBox sequence in their regulatory elements is repressed.

In the first helical region of the STRA8‐HLH domain, there is a basic Nuclear Localization Sequence that might mediate DNA binding to Ebox sequence. However, STRA8 lacks the first glutamate and last arginine residues of the ERXR motif for Ebox recognition.

In the present paper, we aimed to characterize the action of STRA8 as a transcriptional regulator and to investigate whether its HLH domain, by mediating the interaction with others HLH protein/s including the germ cell specific bHLH factor SOHLH1 could modulate their transcriptional function.

## 2 MATERIALS AND METHODS

### 2.1 Cell culture

HEK293T cells and P19 Embryonal Carcinoma (EC) cells (ATCC) were grown in Dulbecco's modified Eagle's medium (DMEM‐ High glucose) with 1% penicillin and streptomycin, 2 mmol/L L‐Glutamine, 0.1 mmol/L non‐essential amino acid (Sigma‐Aldrich), and 10% foetal bovine serum (FBS) (Lonza) under standard culture conditions.

### 2.2 Male and female germ cells isolation

Postnatal male germ cells were obtained from 10 days *post‐partum* (dpp) CD1 albino mice as reported.^23^ When indicated, separation of KIT‐positive from KIT‐negative male germ cells was performed by magnetic‐activated cell sorting (mini‐MACS) with CD117 conjugated microbeads (Miltenyi Biotech) as previously described.^23^ PGCs from 12.5 dpc ovaries were purified using the Mini‐MACS immunomagnetic sorting method.^24^ Female germ cells were isolated from 14.5 and 16.5 days *post‐coitum* (dpc) ovaries following digestion for 10 minutes in Trypsin/EDTA solution (Lonza) and mechanical disaggregation in a monodispersed cell suspension. The cells were cultured for 30 minutes at 37°C and 5% CO_2_ in air in DMEM and samples of cells remaining in suspension, roughly consisting of 70%‐80% of oocytes, were collected for RNA preparation.

### 2.3 DNA constructs

Plasmids expressing the fusion protein GFP‐STRA8 and Myc‐STRA8 were constructed as reported.^15^ pcDNA3‐STRA8 and GST‐STRA8 was obtained by subcloning the coding sequence of mouse *Stra8* by EcoRI/XhoI digestion of pcDNA3‐Myc‐*Stra8* within pcDNA3.1 vector (Promega) and the C‐terminus of pGEX‐4T (GE Healthcare) by using restriction enzymes EcoRI and SalI. The deletion mutants GST‐HLH‐only‐STRA8, Myc‐HLH‐only‐STRA8 (aa 1‐84), GST‐ΔHLH‐STRA8 and Myc‐ΔHLH‐STRA8 (aa 99‐393) were obtained amplifying corresponding fragments by using pcDNA3‐Myc‐*Stra8* as template and primers (1‐4) as indicated in Table 1. For c‐*Kit* promoter construct, (pGL3‐CMV‐*Kit*‐LUC), a 2149 bp fragment (−2525 to −376) of mouse *Kit* promoter (the sequence was derived from EPD Eukaryotic promoter database at https://epd.epfl.ch/mouse/mouse_database.php?db=mouse), was amplified by P19EC cells genomic DNA using primers listed in Table 1 and ligated to the XhoI and NcoI sites of pGL3‐CMV‐LUC vector (gently provided by Prof. C. Sette). The coding sequence of mouse *Sohlh1* (GeneBank NM_001001714), were amplified by PCR from total RNA obtained from P10 male germ cells using primers listed in Table 1 and cloned in BamH1/XhoI sites of pcDNA3 vector. The sequences of the vectors were verified by DNA sequencing (BMR Genomics, Italy). pcDNA3‐Flag‐E47 was generously provided by Prof. Y. Yokota (University of Fukui, Japan). pGEX‐E47 was obtained by Prof. Pura Munoz (Pompeu Fabra University, Barcelona), while V. Saccone (Centro Europeo Ricerca sul cervello, CERC, Rome, Italy) provided pHA‐MYOD and pGEX‐MYOD plasmids.

**Table 1:** Sequences of the primers used in this study

1	HLH‐Stra8 fr	AGGAATTCATGGCCACCCCTGGAG
2	HLH‐Stra8 rev	AGGTCGAGCTTATCCAGCTTTCTTCC
3	ΔHLH‐Stra8 fr	AGGAATTCCCCAACAGCTTAGAGGA
4	ΔHLH‐Stra8 rev	AGGTCGAGTTACAGATCGTCAAAG
5	c‐KIT‐LUC‐fr	AGCTCGAGCACTTCTGGAGATGCTATCTT
6	c‐KIT‐LUC‐rev	AGTCCATGGTAAAGGACAACCACCGGTCC
7	Myc‐Sohlh1 fr	AGGAATCATGGCGTCCGGTGGTG
8	Myc‐Sohlh1 rev	AGGTCGAGTCAGGGGAAAAAGTCA
14	*E47* for	ACAGATGAGGTGCTGTCCCTG
15	*E47* rev	TCACAGGTGCCCAGCTGGATT
16	*Stra8 fr*	GTTCCTGCGTGTTCCACAAG
17	*Stra8 rev*	CACCCGAGGCTCAAGCTTC
18	*Gapdh* for	AACTTTGGCATTGTGGAAGG
19	*Gapdh* rev	CCGTGTTCCTACCCCCAATGTG
20	*Kit fr*	GAGACGTGACTCCTGCCATC
21	*Kit rev*	TCATTCCTGATGTCTCTGGC
22	*L34 fr*	GGTGCTCAGAGGCACTCAGGATG
23	*L34 rev*	GTGCTTTCCCAACCTTCTTGGTGT

### 2.4 Cell transfection

HEK293T cells were transiently transfected with the different plasmids by using the jetPei ^TM^ Polyplus transfection reagent (Polyplus–Transfection SA, SIC, Italy), according to the manufacturer's protocol. 2.5 × 10^6^ P19EC cells were electroporated with 3 µg total DNA by using Cell Line Nucleofector kit and AMAXA nucleofector device II (C‐20 program) (Amaxa).

### 2.5 Immunofluorence analysis

For immunohistochemistry, serial 6 μm thick sections were obtained from testes of 10 dpp mice, fixed in buffered formalin and paraffin embedded. Slides were dewaxed, rehydrated and microwaved in 10 mmol/L sodium citrate buffer, pH 6 for 20 minutes. After blocking with 10% goat serum (GS), sections were incubated with rabbit polyclonal anti‐STRA8 (1:400 Ab49405, Abcam) or rabbit polyclonal anti‐E47 antibodies (1:200 N‐649 sc763, Santa Cruz Biotechnology) diluted in PBS/0.1% BSA/0.1% Triton at 4°C overnight. After washing with PBS/0,5% Tween, 1:400 goat anti‐rabbit (Alexafluor 568, Thermo Fisher Scientific Inc) were used as secondary antibodies for 1 hour incubation at RT. Hoechst in PBS was added for 5 minutes and the samples were observed under a TCS SP5 laser‐scanning confocal microscope (LEICA microsystem, Switzerland). For HA‐MYOD and FLAG‐E47 immunolocalization in HEK293T, cells attached to poly‐L‐lysine‐coated coverslips were transfected as indicated and 24 hours after transfection cells were fixed with 4% (v/v) paraformaldehyde for 10 minutes. The cells were permeabilized with 0.1% Triton X‐100 in PBS for 5 minutes at RT and after blocking with 10% GS for 45 minutes, the anti‐HA antibody (1:500, MMS‐101P Covance) and the anti‐FLAG antibody (1:500, F3165 Sigma‐Aldrich) were applied for 1 hour at RT. After washing with PBS, cells were incubated with goat anti‐mouse antibodies (Alexa Fluor, Molecular Probes) for 45 minutes and with Hoechst for 5 minutes. The samples were observed under a Leica CTR600 microscope with a 40X objective.

### 2.6 Immunoprecipitation

Transfected HEK293T cells, P10 dpp‐isolated male germ cells or P19 EC cells cultured with ATRA (1 µmol/L) for 24 hours were lysed (20 minutes at 4°C) in buffer containing 50 mmol/L Tris‐HCl (pH 7.6), 150 mmol/L NaCl, 2 mmol/L EDTA, 0.5% NP‐40, 3% glycerol, 10 μg/mL phenylmethylsulfonyl fluoride, and a protease inhibitor mix (Sigma‐Aldrich). When indicated, nuclear extracts were prepared from P10 dpp‐isolated male germ cells by homogenizing cells in hypotonic buffer (10 mmol/L Tris/HCl pH 7.4, 10 mmol/L NaCl, 2.5 mmol/L MgCl_2_, 1 mmol/L DTT, protease inhibitor cocktail (PIC) (Sigma‐Aldrich). After incubation on ice for 7 minutes, samples were centrifuged at 700 *g* for 7 minutes. Pelleted nuclei were resuspended in hypotonic buffer supplemented with 90 mmol/L NaCl and 0.5% Triton, sonicated and centrifuged (5000 *g* for 15′) on 30% sucrose cushion. 1 mg of proteins were pre‐cleared with Dynabeads protein G (Life Technologies) for 30 minutes then the extracts were incubated with Dynabeads protein G coupled with control IgG or specific primary antibodies for 2 hours at 4°C under rotation. After washing in the same buffer used for the lysis, precipitates were analysed by Western blotting.

### 2.7 Purification of GST fusion proteins

The BL21 strain of *Escherichia coli* was transformed with plasmid pGEX‐STRA8 (full length and corresponding deletions), pGEX‐MYOD or with pGEX‐4T1 for expression of GST and grown at 37°C in LB medium until A_600_ was 0.7, at which time isopropyl‐β‐thiogalactopyranoside (IPTG) was added for 3 hours at a final concentration of 0.5 mmol/L. Bacteria were collected by centrifugation and the pellet was resuspended in GST‐extraction buffer (GEB: 20 mmol/L Tris‐HC,l pH 7.4, 1 mol/L NaCl and 0.2 mmol/L EDTA) with a PIC (Sigma‐Aldrich). The suspensions were sonicated and debris removed by centrifugation. Fusion proteins were affinity purified by adsorption to glutathione‐agarose beads (Sigma‐Aldrich) for 2 hours at 4°C and eluted in the Elution Buffer (EB: 20 mmol/L Glutathione, 100 mmol/L Hepes pH 7.6, 1 mmol/L DTT and 0.1 mmol/L EDTA. Protein concentrations were determined by SDS‐polyacrylamide gel electrophoresis using purified BSA as standard and Coomassie gel staining.

### 2.8 Pull‐down assay

Transfected HEK293T cells and male germ cells were lysed in a pull‐down buffer (PB: 25 mmol/L Tris‐HCl pH 7.5, 150 mmol/L NaCl, 0.5% Triton‐X100, 1 mmol/L EDTA, 5% glycerol and PIC) and protein concentration was determined using a BCA protein assay kit (Pierce). When indicated, GST or GST fusion proteins were cross‐linked to the glutathione‐agarose beads with Dimethyl pimelimidate (DMP, Sigma‐Aldrich. Stock concentration: 13 mg/mL). Briefly, GST protein bound to the GST‐agarose were washed 2 times with 200 mmol/L Triethanolamine pH 8.9 and incubated for 1 hour with the cross‐linking solution (50 mmol/L DMP in 200 mmol/L Triethanolamine pH 8.9). Cross‐link reaction was blocked with 100 mmol/L ethanolamine pH 8.9, and the beads were washed in PBS and used for pull‐down experiments. Cell extracts (500 µg‐1 mg) were pre‐cleared on glutathione‐agarose beads for 1 hour at 4°C, then incubated under constant shaking with GST or GST fusion proteins, that were cross‐linked or not to the beads, for 2 hours at 4°C. After three washes with the PB buffer, adsorbed proteins were eluted in SDS sample buffer and analysed by Western blotting.

### 2.9 Western blot analysis

Cell extracts or immunoprecipitated proteins were diluted in SDS sample buffer and boiled for 5 minutes. Protein was separated on either 10% or 8% SDS‐Page gels and transferred to PVDF Transfer Membrane Hybond^™^ (Amersham Bioscience). Membranes were saturated with 5% non‐fat dry milk in PBS containing 0.1% Tween20 (PBST) for 1 hour at RT. The antibodies (anti ‐c‐MYC sc764, anti‐GFP sc9996, anti‐GST sc9996, anti‐E47 sc763, anti‐GAPDH sc‐32233 were from Santa Cruz Biotechnology; anti‐Flag F3165 was from Millipore; anti STRA8 Ab49405, anti‐SOHLH1 Ab41520 and anti‐HISTONE H3 Ab1791 were from Abcam; anti c‐KIT gently provided by Prof. S. Dolci) were diluted in PBST buffer and added to the PVDF membrane for 1 hour at RT or overnight at 4°C followed by incubation with the appropriate horseradish peroxidase‐conjugated secondary antibodies (Amersham Bioscience) for 45 minutes at RT. The STRA8 and SOHLH1 immunoprecipitated protein were detected after incubation with the peroxidase‐conjugated anti‐rabbit IgG light chain specific (Jackson ImmunoReasearch). All proteins were detected with ECL plus detection reagents (Amersham Bioscience) and visualized by chemiluminescence.

### 2.10 Luciferase reporter assay

4 × 10^4^ HEK293T cells or P19 EC cells were seeded in 24 wells and transfected with 200 ng of the LUC reporters. The p4RE‐LUC (Dr V. Saccone, (Centro Europeo Ricerca sul cervello, CERC, Rome, Italy), consists of 4 copies of right EBox from *Muscle Creatine Kinase* (MCK) enhancer.^25^ AP1, RARE and 5XCRE‐DNA‐binding sequences were gently provided by Prof. C. Sette, Catholic University of the Sacred Heart, Rome, Italy; Dr D. Barettino, Instituto de Biomedicina de Valencia, Valencia, Spain and Dr PJS Stork, Vollum Institute, Portland, respectively. pGL3‐CMV‐*Kit*‐LUC was described before. Each well also received 10 ng of a pRL‐TK Vector (Promega) to normalize for transfection efficiency. At 48 hours after transfection, cells were washed three times with PBS and scraped in 100 µL of reporter lysis buffer (Promega). Luciferase activity in 20 µL of the cell extracts was quantified using the Dual‐Luciferase Reporter assay system (Promega). Each extract was assayed three times with a Hidex luminometer (RadTech, Italy). The Firefly luciferase activity was divided by Renilla luciferase activity and the transcriptional effect expressed as relative activity compared to the control groups.

### 2.11 Total RNA isolation, RT‐PCR and qRT‐PCR

Total RNA was extracted from the cells using Trizol reagent (Invitrogen) following manufacturer instructions. 1 µg of RNA was retrotranscribed using M‐MLV reverse transcriptase (Promega). 25 ng of cDNA was used as template for PCR (GoTaq, Promega) and reactions were analysed on agarose gels. For quantitative RT‐PCR (qPCR) experiments, SYBR green PCR master mix (Kapa Biosystem) was used following manufacturer's instructions. L34 gene expression was used for normalization in qPCR experiments All primers used in these experiments were indicated in Table 1.

### 2.12 Statistical analyses

All experiments were replicated at least three times. Data were expressed as mean ± Standard Deviation (SD). Student's *t*‐test and one‐way ANOVA analysis were performed using Graphpad Prism software.

## 3 RESULTS

### 3.1 STRA8 interacts with itself in vitro and in vivo through the HLH domain

**FIGURE 1:**
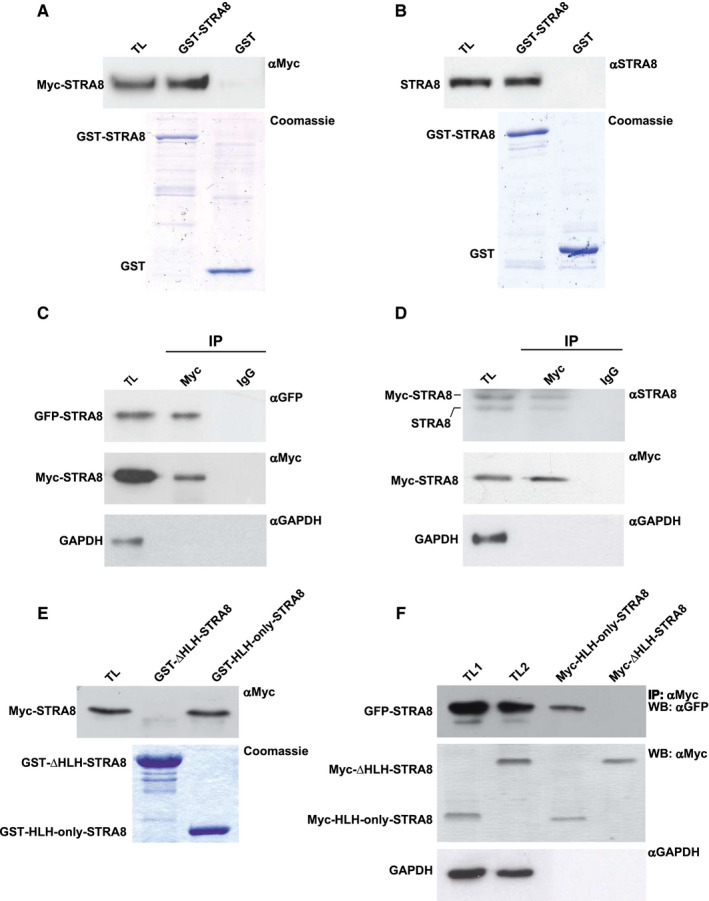
STRA8 interacts with itself through its HLH domain. A‐B, GST pull‐down assay. GST and GST‐STRA8 fusion proteins were incubated with total lysates (TL) obtained from pcDNA3‐Myc‐*Stra8*‐transfected HEK293T cells (A) or male germ cells isolated from P10 dpp testis (B). Bound proteins were visualized by Western blotting with anti‐Myc and anti‐STRA8 antibodies. Coomassie staining shows the purified fusion proteins used in the assay (lower panels). C‐D, Interaction of STRA8 in intact cells. HEK293T cells were co‐transfected with pcDNA3‐Myc‐*Stra8* and pEGFP‐*Stra8* expression vectors (C) while P19EC cells were transfected with pcDNA3‐Myc‐*Stra8* and treated for 24 h with 1 µmol/L ATRA (D). Cell lysates (TL) were immunoprecipitated with anti‐Myc antibody and immunoblotted with anti‐GFP (C), anti‐STRA8 (D), anti‐Myc and anti‐GAPDH antibodies (C,D). E‐F, HLH domain mediates STRA8 interaction. E, Pull‐down assay. GST‐HLH‐only‐STRA8 and GST‐ΔHLH‐STRA8 were incubated with lysates obtained from HEK293T cells (TL) transfected with pcDNA3‐Myc‐*Stra8*. Western blot analysis was performed with anti‐Myc antibody and the amount of purified GST fusion proteins used in the assay was determined by Coomassie staining (lower panel). F, Co‐immunoprecipitation assay. HEK293T cells were co‐transfected with pEGFP‐*Stra8* and the mutants pcDNA3‐Myc‐HLH‐only‐*Stra8* or pcDNA3‐ΔHLH‐*Stra8*. Cell total lysates obtained from the two different experimental conditions (TL1: total lysates from cells over‐expressing GFP‐STRA8 and Myc‐only‐STRA8; TL2: total lysate from cells over‐expressing GFP‐STRA8 and Myc‐ΔHLH‐STRA8) were immunoprecipitated with anti‐Myc antibody and immunoblotted as indicated

Since STRA8 possesses a well conserved HLH domain (aa 17‐84) that could mediate protein‐protein interaction, we tested the ability of the protein to interacts with itself. GST and GST‐STRA8 fusion proteins were used in pull‐down assays with lysates obtained from HEK293T transfected with pcDNA3‐Myc‐STRA8 or from 10 dpp male germ cells. As shown in Figure 1, both recombinant (1A) and endogenous (1B) STRA8 were able to specifically bind to GST‐STRA8 and not to GST alone, thus indicating that the protein efficiently interacts in the in vitro assay. We further investigated whether STRA8 was able to form complexes in intact cells by a co‐immunoprecipitation (CO‐IP) assay. Efficient co‐precipitation of transfected Myc‐STRA8 with GFP‐STRA8 (Figure 1C) and endogenous STRA8 (Figure 1D) was observed. To verify if HLH domain was involved in the interaction, we generated two deletion mutants of STRA8, one consisting of the first 84 aminoacids (Myc‐HLH‐only‐STRA8, GST‐HLH‐only‐STRA8) and another in which the HLH domain was deleted (aa 99‐393, Myc‐ΔHLH‐STRA8, GST‐ΔHLH‐STRA8). We repeated GST pull‐down and co‐immunoprecipitation assays with these mutants. Lysates obtained from HEK293T cells transfected with pcDNA3‐Myc‐STRA8 were used in pull‐down assays with the fusion proteins GST‐HLH‐only‐STRA8 or GST‐ΔHLH‐STRA8 and as shown in Figure 1E, the interaction in vitro was evident only with the HLH‐only‐containing mutant. HEK293T cells were transfected with pEGFP‐STRA8 and pcDNA3‐Myc‐HLH‐only‐STRA8 or pcDNA3‐Myc‐ΔHLH‐STRA8 and after 24 hours of culture lysates were immunoprecipitated with an anti‐MYC antibody. As shown in Figure 1F, deletion of the HLH domain of STRA8 resulted in a complete loss of the interaction with the full length GFP‐STRA8, thus indicating that this STRA8’s domain is involved in the interaction.

### 3.2 STRA8 interacts in vitro and in vivo with class I bHLH E47 through its HLH domain

**FIGURE 2:**
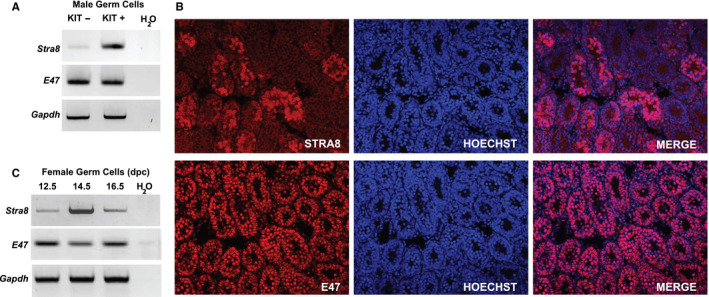
E47 expression in postnatal male germ cells and embryonic female germ cells. A, RT‐PCR analysis of *Stra8*, *E47* and *Gapdh* (as a control) expression in germ cells isolated from P10 dpp testis. Undifferentiated KIT^‐^ and differentiating KIT^+^ cells were purified through immunomagnetic sorting as indicated in Materials and Methods. B, Immunodetection of STRA8 and E47 in histological adjacent sections from 10 ddp mouse testis. C, RT‐PCR analysis of *Stra8*, *E47* and *Gapdh* (as a control) expression in female germ cells obtained from ovaries at the indicated embryonal ages

Tissue specific bHLHs transcription factors are known to form homo and heterodimers with class I bHLH factors through their HLH domain and this is important for efficient DNA binding. With the aim to identify bHLH transcription factors with which STRA8 interacts in germ cells, we concentrated on E proteins, a class of widely expressed bHLH partners. In particular, we investigate *Tcf3/E47* gene, a splicing product of the *Tcf2a* gene that is known to be involved in multiple differentiation processes.^26^ Single cell RNA‐sequencing analysis from mouse testis in the perinatal period^27^ or from sorted KIT‐negative and KIT‐positive cells,^28^ indicated that *E47* is expressed in both spermatogonia populations. Moreover, this gene is also expressed in the preleptotene cells.^13^ To verify the expression of *E47* in male germ cells, we performed RT‐PCR with total RNA obtained from immunomagnetic‐purified undifferentiated (KIT‐negative) and differentiating (KIT‐positive) P10 dpp germ cells. As shown in Figure 2A, we confirmed that *E47* was expressed in both cell populations. As a control for the enrichment of the cell separation, *Stra8* expression was evaluated, confirming its presence prevalently in the KIT‐positive germ cell population^23^, ^29^ (Figure 2A). Immunolocalization of STRA8 in the testis from P10 mice showed an uneven distribution of the protein in different tubules as expected^8^ with spermatogonia and preleptotene positive cells (Figure 2B). E47, on the other hand, is expressed in the nuclei of the cells in each tubule thus indicating that STRA8‐positive cells also express the bHLH factors at this age. RT‐PCR performed in pre‐meiotic (12.5 dpc) and meiotic (13.5‐14.5 dpc) female germ cells obtained by mouse embryos, showed that *E47* transcript was detected in all female germ cell samples analysed (Figure 2C).

**FIGURE 3:**
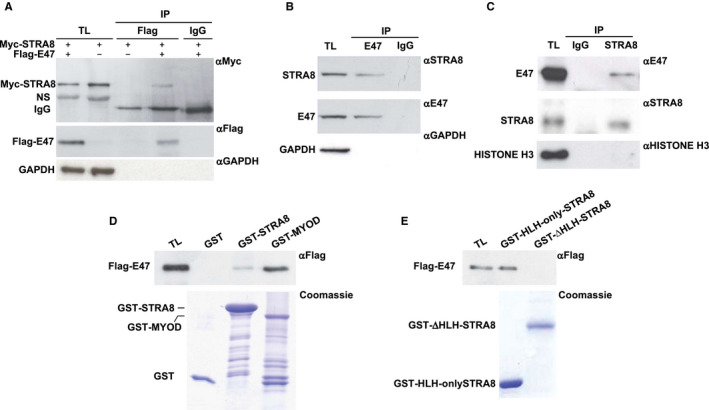
STRA8 interaction with E47 in vivo and in vitro. A, Western blot analysis with anti‐Myc, anti‐FLAG and anti‐GAPDH antibodies of the immunoprecipitation assay performed with control mouse IgG or anti‐FLAG antibody of total lysates (TL) of HEK293T over‐expressing FLAG‐E47 and pcDNA3‐Myc‐STRA8. B‐C, Co‐immunoprecipitation assay for endogenous E47 and STRA8. B, TLs obtained from P10 dpp germ cell suspension were incubated with anti‐E47 or rabbit IgG as a control. Immunoprecipitated proteins were analysed by Western blot using anti‐STRA8, anti‐E47 and anti‐GAPDH antibodies. C, Proteins from nuclear extracts obtained from the same cell suspension were immunoprecipitated with anti‐STRA8 antibody and E47, STRA8 and HISTONE H3 were evaluated by Western blotting. D, Western blot analysis for Flag‐E47 in pull‐down assays of TL of HEK293T cells expressing Flag‐E47 with fusion proteins GST‐STRA8 and GST‐MYOD (positive control). GST was used as negative control. Coomassie staining shows the purified GST and GST fusion proteins (lower panels). E, Pull‐down assay was repeated using the same TLs probed with GST‐HLH‐only‐STRA8 or GST‐ΔHLH‐STRA8 and analysed by Western blot with anti‐Flag antibody. The amount of GST fusion proteins used in the assay was showed in a Coomassie staining of the gel

By using the co‐immunoprecipitation assay, we next investigated whether STRA8 and E47 interacted. A Myc‐tagged *Stra8* expression vector was transfected into HEK293T cells together with pcDNA3‐E47‐Flag using a Flag‐empty vector as a control and total extracts were subjected to immunoprecipitation with an anti‐FLAG antibody. The results in Figure 3A showed that STRA8 was co‐immunoprecipitated with E47 and the specificity of the immunoprecipitation was confirmed with an isotype‐matched nonspecific mouse IgG. Co‐immunoprecipitation performed using total lysates from P10‐isolated male germ cells, that include both STRA8 and E47 expressing cells (Figure 2B) confirmed that the anti‐E47 antibody co‐immunoprecipitated STRA8 also in these cells (Figure 3B). The same result was obtained when anti‐STRA8 antibody was used to immunoprecipitate E47 from nuclear extracts of the germ cells (Figure 3C).

To analyse which domain of STRA8 was important for the interaction with E47, we performed a GST pull‐down assay. Cross‐linked GST‐STRA8 and GST‐MYOD fusion proteins were used for interaction with Flag‐E47 that was transiently transfected in HEK293T cells. GST protein was used as a negative control. As shown in Figure 3D, GST‐STRA8 as GST‐MYOD specifically interacted in vitro with E47 whereas the control GST protein exhibited no interaction. When this assay was repeated with the deletion mutants GST‐HLH‐only‐STRA8 and GST‐ΔHLH‐STRA8 fusion proteins, only the mutant with the HLH domain formed a specific complex with E47 (Figure 3E), thus indicating that this region mediates also the interaction of STRA8 with the bHLH factor.

### 3.3 STRA8 inhibits bHLH‐dependent transcriptional activity through its HLH domain in transfected cells

**FIGURE 4:**
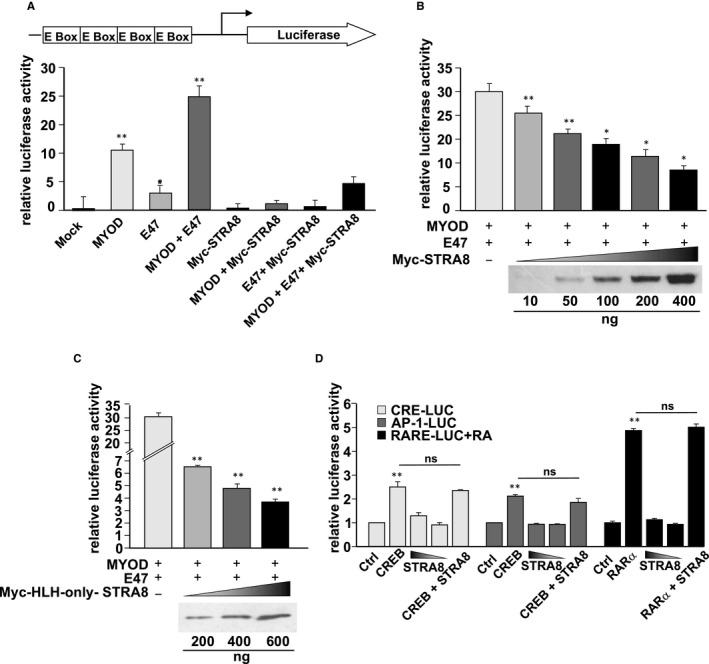
STRA8 inhibits EBox‐mediated transcription. A, HEK293T cells were transiently transfected with *MyoD*‐, *E47*‐ and *Stra8*‐expression plasmids (200 ng) in the indicated different combinations and with the EBox‐mediated luciferase reporter. After 48 h of culture, *Firefly* luciferase expression was evaluated and transfection efficiency normalized by *Renilla* luciferase. The results were expressed as relative luciferase activity setting that of the 4RE‐Luc vector as 1 (mean ± SD, *P < 0.05, **P < 0.01 compared to mock: §§*P* < 0.01compared to MYOD; #*P* < 0.05 compared to E47). B, pHA‐*MyoD* and pcDNA3‐*E47*‐Flag were co‐transfected in HEK293T cells with increasing amounts of pcDNA3‐Myc‐*Stra8* and luciferase assays were performed as above (mean ± SD, *P < 0.05, **P < 0.01). C, Different concentrations of Myc‐HLH‐only‐*Stra8* deletion mutant were analysed in the same EBox‐mediated luciferase assay system (mean ± SD, **P < 0.01). D, Specificity of the STRA8 inhibitory action. HEK293T cells were transiently transfected with the indicated reporters (200 ng) in which luciferase transcription was driven by AP1, CRE and RARE elements. A pcDNA3‐Myc‐*Stra8* vector was added as indicated. For AP1 and CRE‐mediated transcription, an expression vector for CREB was used as a positive control. For RARE activity stimulation, the cells were transfected with a RARγ expression plasmid and treated for 24 h with 1 µmol/L ATRA. (mean ± SD, **P < 0.01 compared to Ctrl; ns: not significant)

Although STRA8 is described as a HLH‐transcriptional regulator,^9^, ^13^, ^15^ in the literature it is not clear if the HLH domain is involved in its transcriptional activity, also because when the HLH region was deleted or muted, STRA8 did not localize to the nucleus.^13^, ^15^ Moreover, STRA8 did not bind the regulated gene promoters to the canonical EBox motif recognized by the tissue specific bHLH transcription factors.^13^, ^14^ Performing a sequence alignment of STRA8 and different bHLH factors, including those expressed in germ cell (MEIOSIN, SOHLH1, SOHLH2) with Clustal Omega program (https://www.ebi.ac.uk), we verified that its basic N‐terminal region did not actually included the determinants for the EBox recognition^19^, ^20^ (Figure S1). Therefore, we hypothesize that STRA8, binding to bHLH factors might negatively modulate their action as the basic region‐lacking ID protein do.^21^ To test this, we investigated if STRA8 was able to repress the activity of an EBox reporter stimulated by two bHLH proteins such as MYOD and E47. Transient transfection experiments were performed in HEK293T using the 4RE‐LUC reporter and vectors expressing STRA8, E47 and MYOD in different combinations as indicated. As shown in Figure 4A, both MYOD and E47, alone or in combination, were able to significantly stimulate the luciferase transcription, while STRA8 failed to induce the transactivation above the basal level. Conversely, when STRA8 was co‐expressed with E47 and MYOD, a significant dose‐dependent inhibitory effect on luciferase transactivation induced by E47 and MYOD homodimers and E47/MYOD heterodimers was observed (Figure 4A,B). The reporter used in these assays consists of four copies of Ebox from *Muscle Creatin Kinase* (*Mck*) enhancer and it has been shown that the heterodimer MYOD/E47 is more efficient in the trans‐activation.^30^ For this reason, we hypothesized that STRA8 inhibited the MYOD‐induced stimulation of reporter activity by interfering with the complex formed by MYOD and endogenous E47. Immunolocalization of E47 and MYOD in transfected HEK293T cells indicated that the two TFs were localized in the nucleus both in the presence and in absence of STRA8‐expressing vector (Figure S2). To determine whether the inhibitory activity of STRA8 was related to the HLH domain, the Myc‐HLH‐only‐STRA8 deletion mutant that localizes in the nucleus^15^ was used in the transcription assay. As shown in Figure 4C, HLH‐only‐STRA8 reduced the stimulation of luciferase activity by E47‐MYOD heterodimers in a dose‐dependent manner.

To investigate whether the inhibitory effect of STRA8 was specific on EBox motif, we evaluated the activity of over‐expressed Myc‐*Stra8* in reporter transactivation experiments in which luciferase was under the control of different DNA‐binding motifs. As shown in Figure 4D, STRA8 did not affect the basal level of each luciferase reporter and did not interfere with the stimulatory activity of CREB through the AP1 or CRE elements and of RARα through the RARE responsive elements.

### 3.4 STRA8 interacts with the germ cell specific bHLH factor SOHLH1 and represses its induced c‐KIT expression in vitro

**FIGURE 5:**
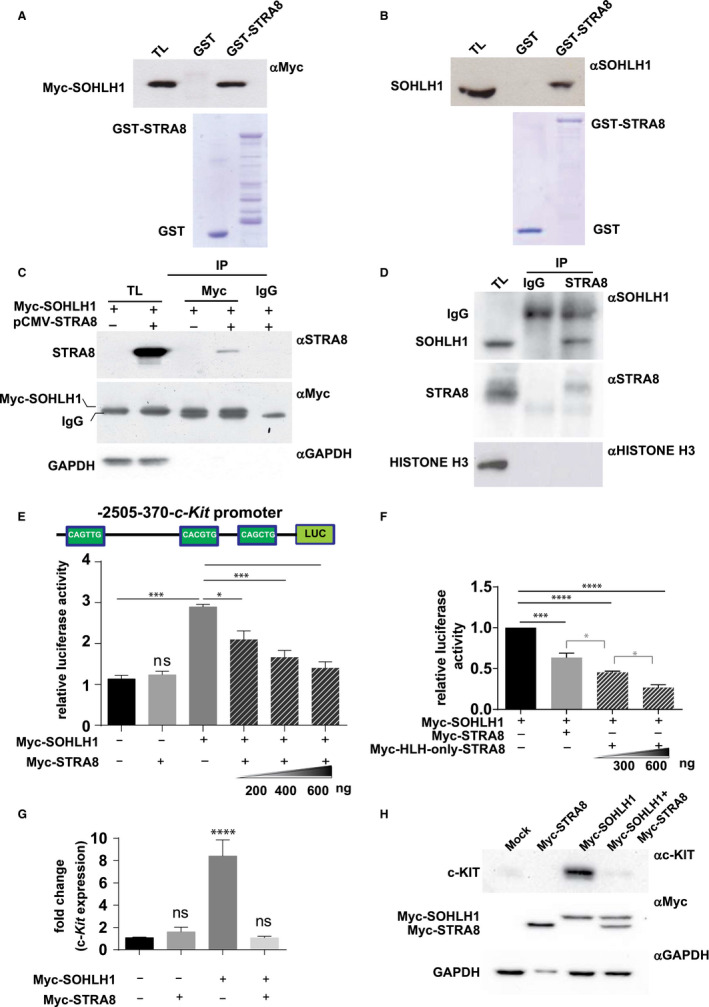
STRA8 interacts with SOHLH1 and down‐regulates SOHLH1‐induced c‐KIT expression in vitro. A‐B, Western blot analysis with anti‐Myc (A) and anti‐SOHLH1 (B) antibodies of the pull‐down assays performed with total lysates (TL) from HEK293T expressing pcDNA3‐Myc‐SOHLH1 (A) or from P10 dpp‐isolated male germ cells and fusion proteins GST‐STRA8 and GST (as negative control). The amount of purified GST fusion proteins used in the assay was determined by Coomassie staining (A,B lower panels). C, Western blot analysis with anti‐STRA8, anti‐Myc and anti‐GAPDH antibodies of the immunoprecipitation performed with control mouse IgG or anti‐Myc antibody of total lysates (TL) of HEK293T expressing pcDNA3‐Myc‐SOHLH1 and/or pCMV‐STRA8. D, The co‐immunoprecipitation assay was repeated with nuclear extracts obtained from P10 dpp‐isolated male germ cells. SOHLH1 immunoprecipitated by anti STRA8 antibody was revealed in Western blot with anti SOHLH1 antibody (upper panel) and as a control of immunoprecipitation, the membranes was incubated with STRA8 and HISTONE H3 antibodies (lower panel). E, The bar graph represents the relative luciferase activity in HEK293T cells transfected with c‐Kit promoter reporter in combination or not with pcDNA3‐Myc‐SOHLH1 and/or different amount of pcDNA3‐Myc‐STRA8. A schematic representation of the Eboxes in the c‐Kit promoter is showed. Data represent mean ± SD of three replicates. *P < 0.05; ***P < 0.001; ns, not significant related to control (ordinary one‐way ANOVA, Tukey's multiple comparisons test). F, Different concentrations (300 and 600 ng) of Myc‐HLH‐only Stra8 mutant were analysed in the same EBox‐mediated luciferase assay (mean ± SD, ***P < 0.001; ****P < 0.0001). G‐H, qPCR (G) and Western blot (H) analyses of c‐KIT expression in P19EC cells transfected with Myc‐STRA8 and/or Myc‐SOHLH1 vectors or control plasmid (mock). G, Fold change of c‐Kit expression relative to Gapdh expression was calculated by the ΔΔCq method. Data represent mean ± SD of three replicates. ****P < 0.0001; ns, not significant (ordinary one‐way ANOVA, Tukey's multiple comparisons test). H, Western blot analysis of c‐KIT expression in P19EC cells transfected as above. Over‐expression of recombinant proteins was evaluated with anti‐Myc antibodies. GAPDH was evaluated as loading control

SOHLH1 and SOHLH2 (SPERMATOGENESIS‐ AND OOGENESIS‐SPECIFIC BHLH TRANSCRIPTION FACTOR 1 AND 2) are two germ cell specific bHLH transcription factors that have been recently reported to be essential for spermatogonia^29^, ^30^, ^31^, ^32^, ^33^ and oocyte^34^, ^35^, ^36^ differentiation. SOHLH1 and SOHLH2 are able to form homo‐ and heterodimers.^32^, ^33^ To examine whether STRA8 was able to interact with SOHLH1, we first evaluated the expression of the proteins in the P10 male germ cells suspension including a heterogeneous population of differentiating spermatogonia and preleptotene/early leptotene spermatocytes. As shown in Figure S3, in isolated germ cells, 85% of STRA8‐positive cells were present that expressed different amount of the proteins and 57% of the cells expressing SOHLH1. We then performed GST pull‐down assays incubating GST‐STRA8 fusion protein with total extracts obtained from HEK293T cells transfected with pcDNA3‐Myc‐SOHLH1 or from P10 dpp male germ cells. As shown in Figure 5, GST‐STRA8 strongly interacted in vitro with over‐expressed‐SOHLH1 (Figure 5A) and with endogenous protein (Figure 5B), whereas the control GST protein exhibited no interaction with either proteins (Figure 5A, B). We then performed co‐immunoprecipitation experiments by using total cell extracts obtained from HEK293T cells co‐transfected with STRA8‐ and SOHLH1‐expressing vectors. As showed in Figure 5C, recombinant STRA8 was able to interact with over‐expressed SOHLH1 also in intact cells. The interaction between STRA8 and SOHLH1 was evident also when the co‐immunoprecipitation experiments were repeated immunoprecipitating SOHLH1 from male germ cells nuclear extracts incubated with STRA8 antibody (Figure 5D). It has been demonstrated that SOHLH1 can positively regulate c‐*Kit* expression by binding its promoter at EBox containing sequence.^29^, ^32^ To analyse the effect of STRA8 on such stimulatory action by SOHLH1, we first cloned the c‐*Kit*‐regulatory promoter region (−2525 to −376 bp) including the two EBoxes bound by SOHLH1 in KIT^+^‐spermatogonia^29^ in pGL3‐CMV‐LUC vector. P19EC cells were then transfected with Myc‐SOHLH1, with or without Myc‐tagged STRA8 expression vector, and luciferase activity was measured 48 h after transfection. As shown in Figure 5E c‐*Kit* promoter activity was induced by SOHLH1 construct, while recombinant STRA8 had no effect. Moreover, the co‐transfection of STRA8 with SOHLH1 vector reduced the transcriptional activation in a dose‐dependent manner. The inhibitory activity of STRA8 was related to the HLH domain because when the Myc‐HLH‐only‐STRA8 deletion mutant was used in the transcription assay, it was able to reduce the stimulation of luciferase activity by SOHLH1 in a dose‐dependent manner (Figure 5F).

To verify if STRA8 was also able to negatively modulate the expression of c‐*Kit*, we over‐expressed SOHLH1 in P19EC cells (that expresses low level of c‐*Kit*) with or without Myc‐STRA8. After 24 hours from transfection, we observed that SOHLH1 induced a significant increase of the *c‐Kit* transcript and when STRA8 was co‐transfected with the bHLH factor, it was able to almost completely abolish the *c‐Kit* stimulation by SOHLH1 (Figure 5G). This result was also observed when c‐KIT protein expression was analysed by Western blot analysis (Figure 5H).

## 4 DISCUSSION

In the mouse embryonal ovary and preleptotene spermatocytes a complex transcriptional programme of meiosis beginning regulated by STRA8 has been recently revealed.^13^, ^14^, ^37^ The most part of involved genes appear to be engaged in cell cycle regulation, double strand break repair and chromosome synapsis and most of them are expressed before the beginning of the meiotic prophase 1. Curiously, a significant percentage of the STRA8‐activated genes is not meiosis/germ cells specific. Accordingly, STRA8 might function as ‘amplifier’ of a large transcriptional programme.^13^, ^14^ At the same time, the meiotic programme also includes a significant number of genes; for example, those involved in mitotic cell cycle that must be negatively regulated.^13^, ^14^ Of these, less than 10% showed a direct binding of STRA8 to their promoters.^14^ Data obtained in our laboratory demonstrated that STRA8 is imported into the nucleus of germ cells through a basic NLS located in the N‐terminal HLH domain.^15^ For this reason, such region is important for the regulatory action of STRA8. Moreover, STRA8 possess a transcriptional activation domain in the C‐terminal region of its molecule.^15^, ^16^ The HLH domain is a well‐known protein‐protein interaction domain and the results presented here suggest that STRA8 can interacts with itself through this region. As a rule, in the HLH family of transcription factors, dimerization serves to convert inactive monomeric molecules into transcriptionally active dimeric complexes at specific times during cellular development. The juxtaposition of two basic regions resulting from dimerization forms a DNA‐binding interface able to insert into the major groove in an EBoxes‐specific manner.^19^ Until now, data regarding the transcriptional activity of STRA8 indicated that it is mediated by binding to target site in the genome that doesn't correspond to canonical EBox CANNTG.^13^, ^14^, ^17^ Therefore, the formation of STRA8 complexes could bring in the correct functional conformation the high mobility group (HMG) box domain predicted in the C‐terminal region of the protein^13^ (Appendix1, aa 216‐268) and corresponding to the C‐terminal activation domain.^15^ Compelling data exist suggesting an involvement of numerous HMG‐box proteins in the modulation and maintenance of chromatin structure.^38^ Their action is based on the ability to bend DNA, thus facilitating the binding of other TFs or protein component of the chromatin remodelling machinery.^39^ These characteristics would well adapt to the role hypothesized for STRA8 as amplifier of meiotic programme.

The HLH domain might also mediate the interaction between STRA8 and other transcriptional regulators. One of this is MEIOSIN that, as STRA8, possesses a bHLH domain^14^ and is fundamental for the shift of the cell cycle from mitosis to meiosis. In attempt to identify other STRA8‐interacting HLH factors in gonadal cells, we focused on the widely expressed TCF3/E47. This protein plays a crucial role in the regulation of cell growth and differentiation in a variety of cell lineages.^40^ Its role is achieved by acting as dimerization partner for lineage‐specific HLH proteins.^17^ Until now the involvement of class I bHLH transcription factors in the differentiation of gonadal tissue is documented only for Sertoli cells in which TCF4/E2‐2 interacting with Inhibitor of DNA‐binding (ID) proteins^41^ and TCF3/E47 interacting with SCLERAXIS, regulate the hormonal‐induced gene expression.^42^ It is interesting that the TCF3 EBox binding motif is enriched in the differentiating spermatogonia^27^ and that *Tcf3*/*E47* is among the genes whose transcript is significantly down‐regulated at meiotic initiation in the preleptotene population.^13^ Our data indicate that at P10, E47 is expressed in the nucleus of the germ cells. Thus it is possible to hypothesize that in differentiating spermatogonia and in preleptotene spermatocytes that also express STRA8, the two proteins form a complex functioning in this particular moment of male germ cell differentiation. STRA8, can inhibit the E47‐EBox‐mediated transcriptional activity on artificial promoter and this negative modulatory activity is due to the presence of the N‐terminal HLH domain. Whether this inhibitory action of STRA8 observed in vitro reflects the in vivo situation should await further investigations. It remains also to be addressed which are the downstream target gene/s of E47 regulated in germ cells and possibly involved in their differentiation and/or cell cycle control.

The ability of STRA8 to interfere in vitro with the DNA‐binding is specific towards Ebox motif, since it does not interfere with other transcription factors that bind different motifs (CRE, AP1 or RARE sequences). In this regard, it appears functionally similar to an ID protein that does not bind DNA EBoxes by itself, but by forming non‐functional heterodimer inhibits other activators.^21^ In addition to MEIOSIN^14^ and E47 (this study), here we identify SOHLH1 as other STRA8 interacting bHLH factors. SOHLH1 and SOHLH2 are bHLH transcription factor known to be expressed specifically in the gonads and important for the differentiation of germ cells. SOHLH2 is expressed in embryonal oocytes prior to entry into meiosis^36^ and in KIT‐negative‐undifferentiated spermatogonia.^29^, ^32^, ^35^ In the adult mouse testis, SOHLH1 is prevalently expressed in differentiating KIT‐positive spermatogonia.^29^, ^30^, ^32^ Besides, it resulted expressed also in preleptotene enriched germ cell population^12^ and up‐regulated by ATRA treatment^29^, ^35^ as STRA8 itself.^4^, ^5^ In male spermatogonia, SOHLH1 and SOHLH2 form homo‐ and heterodimers and bind chromatin at EBox sequences upstream of their own genes and other genes which are essential for spermatogonia differentiation, as for example, c‐KIT.^29^, ^32^ The latter, is fundamental in the regulation of the mitotic activity that characterizes the differentiating population of spermatogonia (A1/A2/A3/A4/intermediate/B spermatogonia)^43^, ^44^, ^45^, ^46^ and its expression is dependent by ATRA signalling as SOHLH1 and STRA8.^8^, ^23^, ^47^ It has been hypothesized that the competence to enter meiosis is related to the number of mitotic divisions controlled by the KL/KIT complex. In fact, KIT expression is down‐regulated at the time of meiotic entry in spermatocyte^48^, ^49^, ^50^ as well as in embryonic female PGCs.^50^ In line with the notion that the differentiating spermatogonia are therefore heterogeneous cell populations,^51^, ^52^, ^53^ we previously observed that in prepuberal testis there are cells SOHLH1+/c‐KIT+/STRA8+ (differentiating spermatogonia), but also cells SOHLH1+/c‐KIT‐/STRA8+; this latter population could represent early differentiating spermatogonia or the competent‐late pre‐meiotic spermatogonia.^12^, ^51^ Since *c‐*KIT is important for spermatogonia differentiation and in fact is expressed at high levels in such population, we hypothesize that STRA8 takes part to its negative regulation at the beginning of meiosis interfering with SOHLH1 in late differentiating spermatogonia and preleptotene spermatocytes.

In conclusion, it is possible to postulate that STRA8 is crucial to regulate the spermatogonia cell cycle and differentiation and at the same time for triggering the meiotic programme in different ways. First, with a positive feedback mediated by its direct binding to its own promoter (a consensus STRA8‐binding motif CNCCTCAG is located −782 bp from TSS)^51^ and to the promoter of the positively regulated genes.^12^, ^13^ Moreover, STRA8 is able to modulate negatively genes regulated by bHLH transcription factors by interfering with their DNA recognition capability. The positive and negative modulatory role of a transcriptional regulator is common also to other transcription factors that can activate or repress target genes in different way, depending on a number of factors such as their concentration, the binding/recruitment of additional proteins to the transcriptional complex or following posttranslation modification and not least the epigenetic status of the target cells. The importance of better understanding the c‐KIT and STRA8 regulation is not only related to spermatogenesis, but extend also to testicular germ cell tumours. Alteration as mutations, copy number and expression of c‐KIT and c‐KIT‐PI3K pathway are significantly associated to seminoma.^54^, ^55^ STRA8 and the premature germ cell differentiation are associated to susceptibility to testicular teratomas.^56^, ^57^, ^58^ Therefore, other than for fertility preservation, it is important to understand how STRA8 works to design future therapeutic strategies.

## CONFLICT OF INTEREST

The authors confirm that are not conflicts of interest.

## AUTHOR CONTRIBUTIONS

**Maria Giovanna Desimio:** Conceptualization (equal); Data curation (equal); Formal analysis (equal); Investigation (equal). **Eleonora Cesari:** Formal analysis (equal); Investigation (equal). **Maria Sorrenti:** Data curation (equal); Investigation (equal). **Massimo De Felici:** Conceptualization (equal); Funding acquisition (equal); Supervision (equal); Writing‐original draft (equal). **Donatella Farini:** Conceptualization (equal); Data curation (equal); Formal analysis (equal); Supervision (equal); Writing‐original draft (equal); Writing‐review & editing (equal).

## Supporting information

Fig S1

Fig S2

Fig S3

## Data Availability

All data generated during the study are available from the corresponding author on request.

## References

[1] Rhinn M , Dolle P . Retinoic acid signalling during development. Development. 2012;139:843‐858. 22318625 10.1242/dev.065938

[2] Teletin M , Vernet N , Ghyselinck NB , Mark M . Roles of retinoic acid in germ cell differentiation. Curr Top Dev Biol. 2017;125:191‐225. 28527572 10.1016/bs.ctdb.2016.11.013

[3] Endo T , Mikedis MM , Nicholls PK , Page DC , de Rooij DG . Retinoic acid and germ cell development in the ovary and testis. Biomolecules. 2019;9:775. 10.3390/biom9120775 PMC6995559 31771306

[4] Bouillet P , Oulad‐Abdelghani M , Vicaire S , et al. Efficient cloning of cDNAs of retinoic acid‐responsive genes in P19 embryonal carcinoma cells and characterization of a novel mouse gene, Stra1 (mouse LERK‐2/Eplg2). Dev Biol. 1995;170:420‐433. 7649373 10.1006/dbio.1995.1226

[5] Oulad‐Abdelghani M , Bouillet P , Decimo D , et al. Characterization of a premeiotic germ cell‐specific cytoplasmic protein encoded by Stra8, a novel retinoic acid‐responsive gene. J Cell Biol. 1996;135:469‐477. 8896602 10.1083/jcb.135.2.469 PMC2121034

[6] Anderson EL , Baltus AE , Roepers‐Gajadien HL , et al. Stra8 and its inducer, retinoic acid, regulate meiotic initiation in both spermatogenesis and oogenesis in mice. Proc Natl Acad Sci USA. 2008;105:14976‐14980. 18799751 10.1073/pnas.0807297105 PMC2542382

[7] Menke DB , Koubova J , Page DC . Sexual differentiation of germ cells in XX mouse gonads occurs in an anterior‐to‐posterior wave. Dev Biol. 2003;262:303‐312. 14550793 10.1016/s0012-1606(03)00391-9

[8] Zhou Q , Nie R , Li Y , et al. Expression of stimulated by retinoic acid gene 8 (Stra8) in spermatogenic cells induced by retinoic acid: an in vivo study in vitamin A‐sufficient postnatal murine testes. Biol Reprod. 2008;79:35‐42. 18322276 10.1095/biolreprod.107.066795 PMC3208264

[9] Baltus AE , Menke DB , Hu YC , et al. In germ cells of mouse embryonic ovaries, the decision to enter meiosis precedes premeiotic DNA replication. Nat Genet. 2006;38:1430‐1434. 17115059 10.1038/ng1919

[10] Mark M , Jacobs H , Oulad‐Abdelghani M , et al. STRA8‐deficient spermatocytes initiate, but fail to complete, meiosis and undergo premature chromosome condensation. J Cell Sci. 2008;121:3233‐3242. 18799790 10.1242/jcs.035071

[11] Nagaoka SI , Nakaki F , Miyauchi H , et al. ZGLP1 is a determinant for the oogenic fate in mice. Science. 2020;367:eaaw4115. 32054698 10.1126/science.aaw4115

[12] Endo T , Romer KA , Anderson EL , Baltus AE , de Rooij DG , Page DC . Periodic retinoic acid‐STRA8 signaling intersects with periodic germ‐cell competencies to regulate spermatogenesis. Proc Natl Acad Sci USA. 2015;112:E2347‐2356. 25902548 10.1073/pnas.1505683112 PMC4426408

[13] Kojima ML , de Rooij DG , Page DC . Amplification of a broad transcriptional program by a common factor triggers the meiotic cell cycle in mice. eLife. 2019;8:e43738. 30810530 10.7554/eLife.43738 PMC6392498

[14] Ishiguro KI , Matsuura K , Tani N , et al. MEIOSIN directs the switch from mitosis to meiosis in mammalian germ cells. Dev Cell. 2020;52(4):429–445.e10. 32032549 10.1016/j.devcel.2020.01.010

[15] Tedesco M , La Sala G , Barbagallo F , De Felici M , Farini D . STRA8 shuttles between nucleus and cytoplasm and displays transcriptional activity. J Biol Chem. 2009;284:35781‐35793. 19805549 10.1074/jbc.M109.056481 PMC2791008

[16] Choi Y , Yoon J , Pyo C , Kim J , Bae S , Park S . A possible role of STRA8 as a transcriptional factor. Genes Genom. 2010;32:521‐526.

[17] Massari ME , Murre C . Helix‐loop‐helix proteins: regulators of transcription in eucaryotic organisms. Mol Cell Biol. 2000;20:429‐440. 10611221 10.1128/mcb.20.2.429-440.2000 PMC85097

[18] Jones S . An overview of the basic helix‐loop‐helix proteins. Genome Biol. 2004;5:226. 15186484 10.1186/gb-2004-5-6-226 PMC463060

[19] Ellenberger T , Fass D , Arnaud M , Harrison SC . Crystal structure of transcription factor E47: E‐box recognition by a basic region helix‐loop‐helix dimer. Genes Dev. 1994;8:970‐980. 7926781 10.1101/gad.8.8.970

[20] Murre C . Helix‐loop‐helix proteins and the advent of cellular diversity: 30 years of discovery. Genes Dev. 2019;33:6‐25. 30602438 10.1101/gad.320663.118 PMC6317319

[21] Benezra R , Davis RL , Lockshon D , Turner DL , Weintraub H . The protein Id: a negative regulator of helix‐loop‐helix DNA binding proteins. Cell. 1990;61:49‐59. 2156629 10.1016/0092-8674(90)90214-y

[22] Ling F , Kang B , Sun XH . Id proteins: small molecules, mighty regulators. Curr Top Dev Biol. 2014;110:189‐216. 25248477 10.1016/B978-0-12-405943-6.00005-1

[23] Pellegrini M , Filipponi D , Gori M , et al. ATRA and KL promote differentiation toward the meiotic program of male germ cells. Cell Cycle. 2008;7:3878‐3888. 19098446 10.4161/cc.7.24.7262

[24] Pesce M , De Felici M . Purification of mouse primordial germ cells by MiniMACS magnetic separation system. Dev Biol. 1995;170:722‐725. 7649397 10.1006/dbio.1995.1250

[25] Weintraub H , Davis R , Lockshon D , Lassar A . MyoD binds cooperatively to two sites in a target enhancer sequence: occupancy of two sites is required for activation. Proc Natl Acad Sci USA. 1990;87:5623‐5627. 2377600 10.1073/pnas.87.15.5623 PMC54379

[26] Wang LH , Baker NE . E Proteins and ID proteins: helix‐loop‐helix partners in development and disease. Dev Cell. 2015;35:269‐280. 26555048 10.1016/j.devcel.2015.10.019 PMC4684411

[27] Tan K , Song HW , Wilkinson MF . Single‐cell RNAseq analysis of testicular germ and somatic cell development during the perinatal period. Development. 2020;147:dev183251. 31964773 10.1242/dev.183251 PMC7033731

[28] Kubo N , Toh H , Shirane K , et al. DNA methylation and gene expression dynamics during spermatogonial stem cell differentiation in the early postnatal mouse testis. BMC Genom. 2015;16:624. 10.1186/s12864-015-1833-5 PMC4546090 26290333

[29] Barrios F , Filipponi D , Campolo F , et al. SOHLH1 and SOHLH2 control Kit expression during postnatal male germ cell development. J Cell Sci. 2012;125:1455‐1464. 22328502 10.1242/jcs.092593

[30] Weintraub H , Davis R , Tapscott S , et al. The myoD gene family: nodal point during specification of the muscle cell lineage. Science. 1991;251:761‐766. 1846704 10.1126/science.1846704

[31] Ballow D , Meistrich ML , Matzuk M , Rajkovic A . Sohlh1 is essential for spermatogonial differentiation. Dev Biol. 2006;294:161‐167. 16564520 10.1016/j.ydbio.2006.02.027

[32] Hao J , Yamamoto M , Richardson TE , et al. Sohlh2 knockout mice are male‐sterile because of degeneration of differentiating type A spermatogonia. Stem Cells. 2008;26:1587‐1597. 18339773 10.1634/stemcells.2007-0502

[33] Suzuki H , Ahn HW , Chu T , et al. SOHLH1 and SOHLH2 coordinate spermatogonial differentiation. Dev Biol. 2012;361:301‐312. 22056784 10.1016/j.ydbio.2011.10.027 PMC3249242

[34] Toyoda S , Yoshimura T , Mizuta J , Miyazaki J . Auto‐regulation of the Sohlh1 gene by the SOHLH2/SOHLH1/SP1 complex: implications for early spermatogenesis and oogenesis. PLoS One. 2014;9:e101681. 25003626 10.1371/journal.pone.0101681 PMC4086951

[35] Pangas SA , Choi Y , Ballow DJ , et al. Oogenesis requires germ cell‐specific transcriptional regulators Sohlh1 and Lhx8. Proc Natl Acad Sci USA. 2006;103:8090‐8095. 16690745 10.1073/pnas.0601083103 PMC1472434

[36] Toyoda S , Miyazaki T , Miyazaki S , et al. Sohlh2 affects differentiation of KIT positive oocytes and spermatogonia. Dev Biol. 2009;325:238‐248. 19014927 10.1016/j.ydbio.2008.10.019

[37] Shin YH , Ren Y , Suzuki H , et al. Transcription factors SOHLH1 and SOHLH2 coordinate oocyte differentiation without affecting meiosis I. J Clin Investig. 2017;127:2106‐2117. 28504655 10.1172/JCI90281 PMC5451230

[38] Soh YQ , Junker JP , Gill ME , Mueller JL , van Oudenaarden A , Page DC . A gene regulatory program for meiotic prophase in the fetal ovary. PLoS Genet. 2015;11:e1005531. 26378784 10.1371/journal.pgen.1005531 PMC4574967

[39] Stros M , Launholt D , Grasser KD . The HMG‐box: a versatile protein domain occurring in a wide variety of DNA‐binding proteins. Cell Mol Life Sci. 2007;64:2590‐2606. 17599239 10.1007/s00018-007-7162-3 PMC11136187

[40] Malarkey CS , Churchill ME . The high mobility group box: the ultimate utility player of a cell. Trends Biochem Sci. 2012;37:553‐562. 23153957 10.1016/j.tibs.2012.09.003 PMC4437563

[41] Slattery C , Ryan MP , McMorrow T . E2A proteins: regulators of cell phenotype in normal physiology and disease. Int J Biochem Cell Biol. 2008;40:1431‐1436. 17604208 10.1016/j.biocel.2007.05.014

[42] Muir T , Sadler‐Riggleman I , Stevens JD , Skinner MK . Role of the basic helix‐loop‐helix protein ITF2 in the hormonal regulation of Sertoli cell differentiation. Mol Reprod Dev. 2006;73:491‐500. 16425294 10.1002/mrd.20397

[43] Muir T , Sadler‐Riggleman I , Skinner MK . Role of the basic helix‐loop‐helix transcription factor, scleraxis, in the regulation of Sertoli cell function and differentiation. Mol Endocrinol. 2005;19:2164‐2174. 15831523 10.1210/me.2004-0473

[44] Schrans‐Stassen BH , van de Kant HJ , de Rooij DG , van Pelt AM . Differential expression of c‐kit in mouse undifferentiated and differentiating type A spermatogonia. Endocrinology. 1999;140:5894‐5900. 10579355 10.1210/endo.140.12.7172

[45] Rossi P , Sette C , Dolci S , Geremia R . Role of c‐kit in mammalian spermatogenesis. J Endocrinol Invest. 2000;23:609‐615. 11079457 10.1007/BF03343784

[46] Dolci S , Pellegrini M , Di Agostino S , Geremia R , Rossi P . Signaling through extracellular signal‐regulated kinase is required for spermatogonial proliferative response to stem cell factor. J Biol Chem. 2001;276:40225‐40233. 11502745 10.1074/jbc.M105143200

[47] Feng LX , Ravindranath N , Dym M . Stem cell factor/c‐kit up‐regulates cyclin D3 and promotes cell cycle progression via the phosphoinositide 3‐kinase/p70 S6 kinase pathway in spermatogonia. J Biol Chem. 2000;275:25572‐25576. 10849422 10.1074/jbc.M002218200

[48] Busada JT , Chappell VA , Niedenberger BA , et al. Retinoic acid regulates Kit translation during spermatogonial differentiation in the mouse. Dev Biol. 2015;397:140‐149. 25446031 10.1016/j.ydbio.2014.10.020 PMC4268412

[49] Yoshinaga K , Nishikawa S , Ogawa M , et al. Role of c‐kit in mouse spermatogenesis: identification of spermatogonia as a specific site of c‐kit expression and function. Development. 1991;113:689‐699. 1723681 10.1242/dev.113.2.689

[50] Sette C , Dolci S , Geremia R , Rossi P . The role of stem cell factor and of alternative c‐kit gene products in the establishment, maintenance and function of germ cells. Int J Dev Biol. 2000;44:599‐608. 11061423

[51] Sorrentino V , Giorgi M , Geremia R , Besmer P , Rossi P . Expression of the c‐kit proto‐oncogene in the murine male germ cells. Oncogene. 1991;6:149‐151. 1704118

[52] Desimio MG , Campolo F , Dolci S , De Felici M , Farini D . SOHLH1 and SOHLH2 directly down‐regulate STIMULATED BY RETINOIC ACID 8 (STRA8) expression. Cell Cycle. 2015;14:1036‐1045. 25603532 10.1080/15384101.2015.1007721 PMC4614006

[53] Hermann BP , Cheng K , Singh A , et al. The mammalian spermatogenesis single‐cell transcriptome, from spermatogonial stem cells to spermatids. Cell Rep. 2018;25(6):1650‐1667.e8. 30404016 10.1016/j.celrep.2018.10.026 PMC6384825

[54] Green CD , Ma Q , Manske GL , et al. A comprehensive roadmap of murine spermatogenesis defined by single‐cell RNA‐Seq. Dev Cell. 2018;46(5):651‐667.e10. 30146481 10.1016/j.devcel.2018.07.025 PMC6713459

[55] Litchfield K , Summersgill B , Yost S , et al. Whole‐exome sequencing reveals the mutational spectrum of testicular germ cell tumours. Nat Commun. 2015;6:5973. 25609015 10.1038/ncomms6973 PMC4338546

[56] Shen H , Shih J , Hollern DP , et al. Integrated molecular characterization of testicular germ cell tumors. Cell Rep. 2018;23:3392‐3406. 29898407 10.1016/j.celrep.2018.05.039 PMC6075738

[57] Rajpert‐De ME . Developmental model for the pathogenesis of testicular carcinoma in situ: genetic and environmental aspects. Hum Reprod Update. 2006;12:303‐323. 16540528 10.1093/humupd/dmk006

[58] Heaney JD , Anderson EL , Michelson MV , et al. Germ cell pluripotency, premature differentiation and susceptibility to testicular teratomas in mice. Development. 2012;139:1577‐1586. 22438569 10.1242/dev.076851 PMC3317965

