# Supplementary material for: Stimulated by retinoic acid gene 8 (STRA8) interacts with the germ cell specific bHLH factor SOHLH1 and represses c‐KIT expression in vitro
Source: J Cell Mol Med. 2020 Nov 25;25(1):383–96. doi: 10.1111/jcmm.16087 (PMC7810945; doi:10.1111/jcmm.16087)
Supplement: Supplementary file 1 — Fig S1 [file JCMM-25-383-s001.pdf]

```
                                                                    HELIX I               LOOP            HELIX II
STRA8/P70278_1-393    MATPGEGNQP SDDGAPQPLA QLQKLEPRVV RRRLSQARHR ATLVGLFNNL RKAVYSQ... ...SDITASK WQVLNRTKIH IQEQEESLDK LLKLKASFNL
MEIOSIN/D3YZT1_14-68  ---------- ---------- ---------H RARSPSPTDR KDKKNHTNKL RELALLIPVT MKTRDKKYTK KEILLRVLHY IQYLQRNFDM ----------
ID1/P20067_46-98      ---------- ---------- -----LPALL DE.QQVNVLL YDMNGCYSRL KELVPTL... ..PQNRKVSK VEILQHVIDY IRDL------ ----------
ID4/P41139_52-104     ---------- ---------- -----AAEAA AD.EPALCLQ CDMNDCYSRL RRLVPTI... ..PPNKKVSK VEILQHVIDY ILDL------ ----------
SOHLH1/Q6IUP1_54-105  ---------- ---------- -----PSLR. RNVVSERERR RRISLSCEHL RALLPQFDG. ..RREDMAS. VLEMSVYFLQ LLAHSM---- ----------
SOHLH2/Q9D489_200-251 ---------- ---------- ------QASF LHSTKEKLRR ERIKSCCEQL RTLLPYVKG. ..RKSDVAS. VIEATVDYVKQVRE------- ----------
c-MYC/P01108_354-406  ---------- ---------- ------DKRR THNVLERQRR NELKRSFFAL RDQIPELEN. ..NEKAP..K VVILKKATAY ILSI------ ----------
E47/P15806_546-599    ---------- ---------- ------ERRV ANNARERLRV RDINEAFKEL GR.MCQLHL. .SSEKPQT.K LLILHQAVAV ILSLQQVRE- ----------
MYOD/P10085_109-160   ---------- ---------- ------DRRK AATMRERRRL SKVNEAFETL KR.CTSSNP. ....NQRLPK VEILRNAIRY IEGL------ ----------
CONSENSUS Sequence                                R    N  ER R        F  L                     K   IL
                                                         T
```

Figure Supplementary 1
