# Supplementary figures and images for: Stimulated by retinoic acid gene 8 (STRA8) interacts with the germ cell specific bHLH factor SOHLH1 and represses c‐KIT expression in vitro
Source: J Cell Mol Med. 2020 Nov 25;25(1):383–96. doi: 10.1111/jcmm.16087 (PMC7810945; doi:10.1111/jcmm.16087)

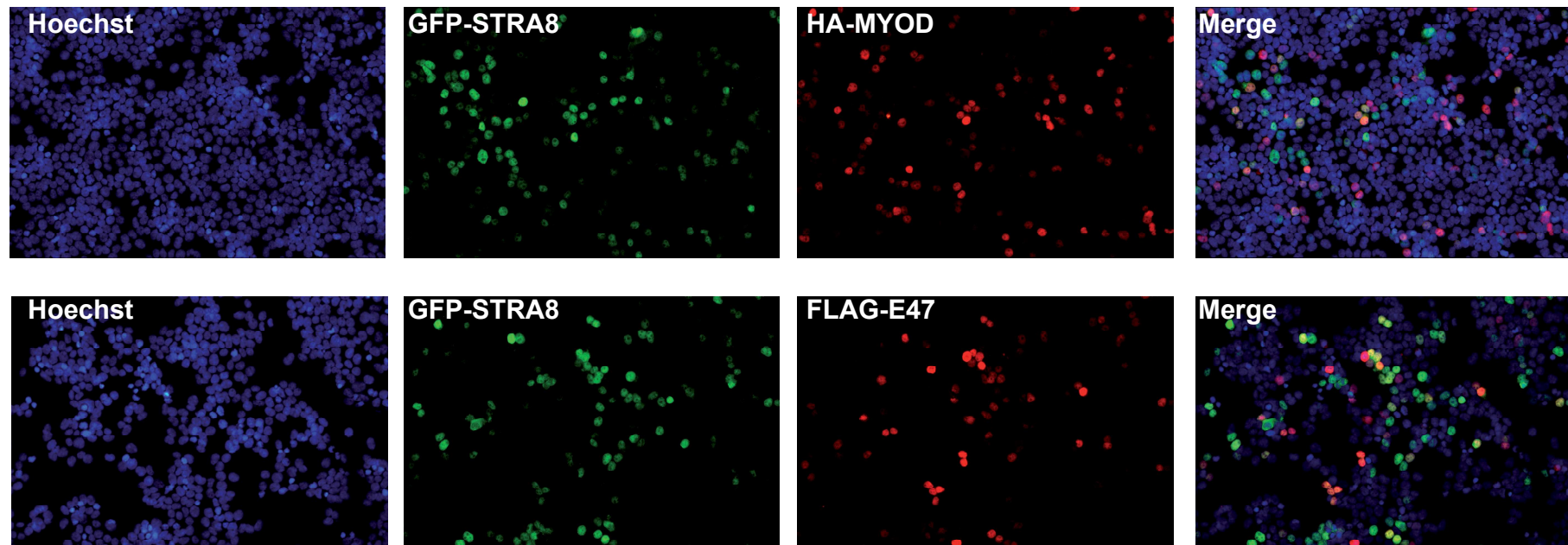

Figure Supplementary 2

Supplement: Supplementary file 2 — Fig S2 [file JCMM-25-383-s002.pdf]

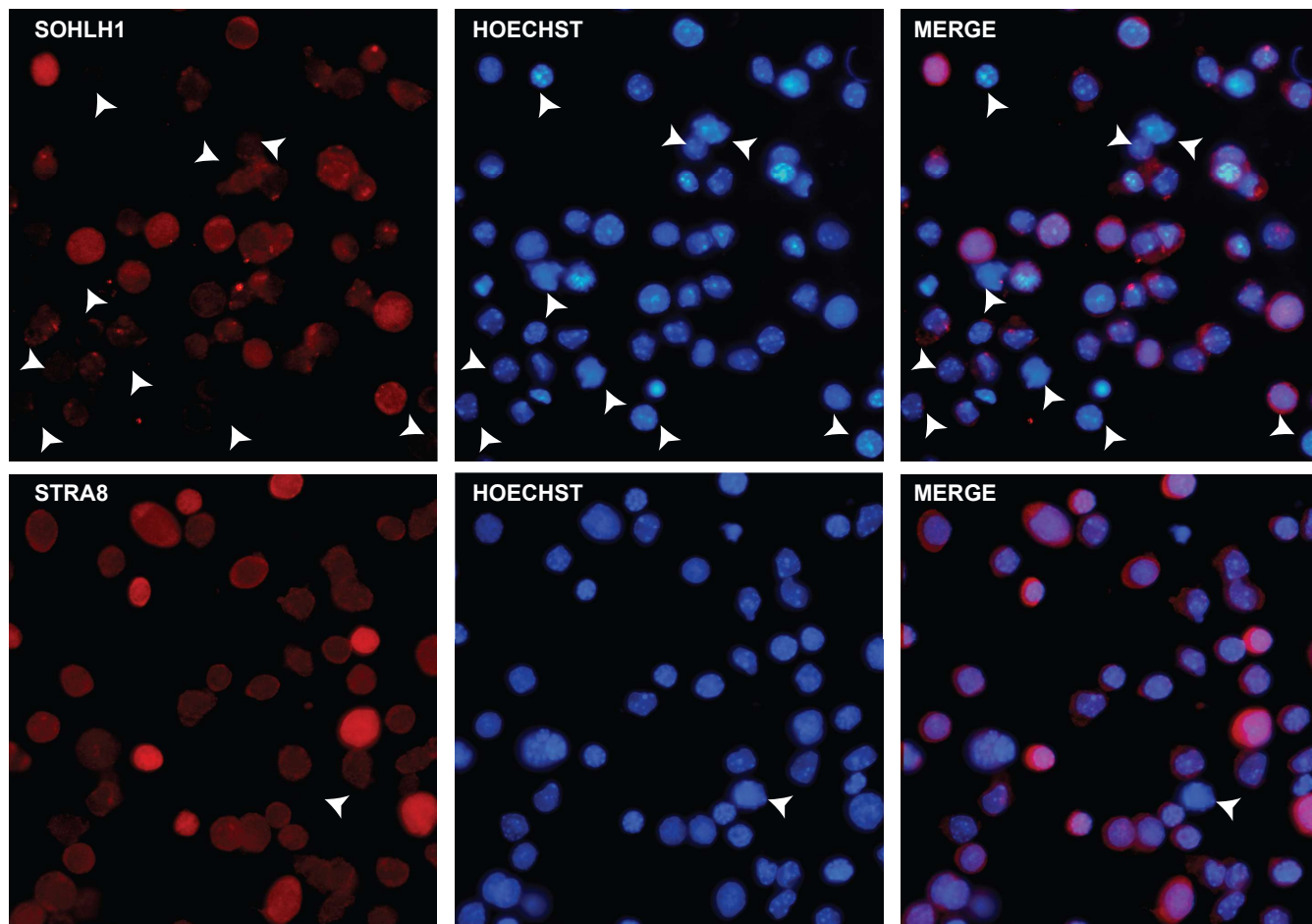

Figure Supplementary 3

Supplement: Supplementary file 3 — Fig S3 [file JCMM-25-383-s003.pdf]
